# Outbreak of Cholera Due to Cyclone Kenneth in Northern Mozambique, 2019

**DOI:** 10.3390/ijerph16162925

**Published:** 2019-08-15

**Authors:** Edgar Cambaza, Edson Mongo, Elda Anapakala, Robina Nhambire, Jacinto Singo, Edsone Machava

**Affiliations:** 1 Department of Biological Sciences, Faculty of Sciences, Eduardo Mondlane University, Av. Julius Nyerere, Maputo nr. 3453, Mozambique; 2 National Health Institute, Distrito de Marracuene, Estrada Nacional N°1, 1120 Maputo Province, Mozambique

**Keywords:** cyclone Kenneth, Mozambique, outbreak, cholera

## Abstract

Cyclone Kenneth was the strongest in the recorded history of the African continent. It landed in the Cabo Delgado province in northern Mozambique on 25 April 2019, causing 45 deaths, destroying approximately 40,000 houses, and leaving 374,000 people in need for assistance, most at risk of acquiring waterborne diseases such as cholera. This short article aims to explain how the resulting cholera outbreak occurred and the response by the government and partner organizations. The outbreak was declared on 2 May 2019, after 14 cases were recorded in Pemba city (11 cases) and the Mecúfi district (3 cases). The disease spread to Metuge, and by the 12th of May 2019, there were 149 cases. Aware of the risk of an outbreak of cholera, the government and partners took immediate action as the cyclone ended, adapting the Cholera Response Plan for Beira, revised after the experience with cyclone Idai (4–21 March 2019). The response relevant to cholera epidemics consisted of social mobilization campaigns for prevention, establishment of treatment centers and units, coordination to improve of water, sanitation and hygiene, and surveillance. By 26 May 2019, 252,448 people were immunized in the area affected by cyclone Kenneth. The recovery process is ongoing but the number of new cases has been reducing, seemingly due to an efficient response, support of several organizations and collaboration of the civil society. Future interventions shall follow the same model of response but the government of Mozambique shall keep a contingency fund to manage disasters such as cyclone Idai and Kenneth. The unlikeliness of two cyclones (Idai and Kenneth) within two months after decades without such kind of phenomena points towards the problem of climate change, and Mozambique needs to prepare effective, proven response plans to combat outbreaks of waterborne diseases due to cyclones.

## 1. Introduction

Mozambique was affected by two tropical cyclones, Idai and Kenneth, and now the country faces aftermath public health challenges [1,2]. Kenneth, a category 3 cyclone (Saffir–Simpson scale [3]), made landfall on the north coast of Mozambique on Thursday, 25 April 2019, at about 13:15 (Coordinated Universal Time), packing wind up to 220 km/h and destroying virtually the entire sanitation infrastructure (sewers, bathrooms and latrines, drainage, etc.), critically increasing the population’s risk of waterborne diseases, particularly in Pemba city and also the Ibo, Macomia and Quissanga districts [4]. Pemba, the province’s capital, is home to approximately 200,000 inhabitants, and it is known as one of the country’s most cholera-endemic areas [5,6].

Cholera was originally introduced to Mozambique from the Indian Subcontinent in 1970, but recent pandemic waves came from other regions worldwide [7]. Since then, there have been regular outbreaks [8]. The country’s northern area—provinces of Nampula, Cabo Delgado and Niassa—is most vulnerable to outbreaks of waterborne diseases [9,10,11,12], which occur annually during the rainy season [8]. The last major outbreak of cholera happened in 2015, with 7073 cases reported and 53 deaths [13]. The following years also had outbreaks but not as widespread and severe, likely due to a robust intervention response integrating immunization, treatment and health education [8,11,14]. However, the cyclone certainly disrupted the annual plan to contain the disease during the rainy season.

The first case of cholera associated with Cyclone Kenneth was observed on 27 April but the Provincial Health Director declared an outbreak on 1 May 2019 after 11 cases were reported in Pemba city and 3 in the Mecúfi district [5,6]. By 8 May 2019, there were 109 cases: 89 in Pemba, 10 in Mecúfi and 10 in Metuge [2]. Authorities were expecting the outbreak, according to the Provincial Director, because of the prior experience with Idai [15,16], responsible for 6743 cholera cases in Mozambique [17]. Furthermore, the National Meteorology Institute had forecasted the cyclone’s landing, and previous experience of regular outbreaks of cholera during the rainy season (due to the heavy rain), led the authorities to expect an outbreak due to Cyclone Kenneth [11,12,15,16].

Since the outbreak started, several risk factors and health determinants indicated that the cholera would likely spread to areas previously unaffected by annual cholera outbreaks. For instance, there was shortage of clean water, damaged sanitation infrastructure, and there were 3527 displaced people living with little access to facilities for personal hygiene [18]. The healthcare system and other infrastructures for assistance of the victims were very limited in these areas [1], even during non-emergency times. In any case, there was, and there is still, need for intervention, and the combined level of devastation by both Idai and Kenneth will certainly take years for recovery. For instance, just for Idai, by 3 April 2019 only $4.6 million (13%) of the US $36.4 million initially requested to assist the victims was available [19,20]. The situation got worse as the amount required escalated to US $282 million [21].

The investigation and response to the cholera outbreak due to cyclone Kenneth are still ongoing as this document is being written, but it is important to keep spreading awareness. The current communication aims to provide some insights on the outbreak of cholera in the north of Mozambique due to cyclone Kenneth, how interventions took place and their impact, and some recommendations on the next steps to follow.

## 2. Sources and Documental Analysis

This study’s protocol was approved by Comité Institucional de Bioética em Saúde da Faculdade de Medicina/Hospital Central de Maputo (CBS FM&HCM) (Institutional Committee of Health Bioethics of the Faculty of Medicine/Central Hospital of Maputo) under the number CIBS FM&HCM/76/2019. The authors granted the maximum confidentiality and anonymity of the information presented in the manuscript.

This short communication was based on a review of the most relevant literature related to the cholera outbreak as result of cyclone Kenneth. The document analysis was performed in ATLAS.ti (ATLAS.ti GmbH, Berlin, Germany) and partly in Microsoft Excel^TM^ (Microsoft, Redmond, Washington, USA), and mainly based on flash updates by the United Nations Office for the Coordination of Humanitarian Affairs (OCHA) [2,4,5,18,22,23], and news from the Cable News Network (CNN) and other agencies [1,6,24].

The first documents analyzed were found through two search engines: Google^TM^ and Google Scholar^TM^. The keywords were “Cyclone Kenneth” and “cholera”. The documents found helped finding other relevant documents. Twenty-one documents resulted from the search. Then, the documents were all introduced in ATLAS.ti, and key epidemiological information was codified in the following categories, adapted from the Public Health Agency of Canada [25]: Context, outbreak identification, process of investigation, interventions and descriptive epidemiology.

## 3. Mozambique

**Figure 1:**
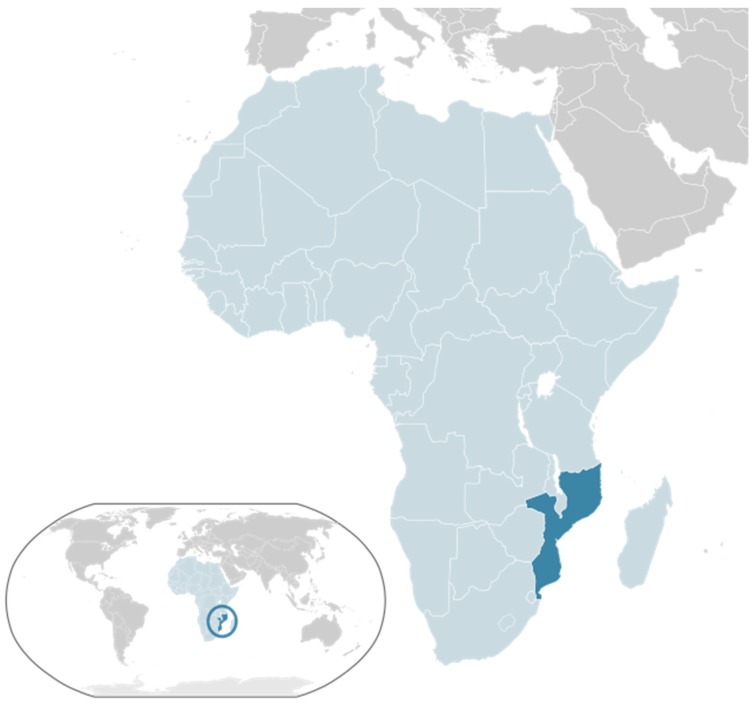
Location of Mozambique on the world map and in Africa. Source: Alvaro [28], released under public domain.

The Republic of Mozambique (Figure 1) is located at the east coast of Southern Africa, with an area of 801,537 km^2^ (25° 57′ S; 32° 35′ E), surrounded by South Africa and Eswatini (southwest), Zimbabwe and Zambia (west), Malawi (northwest) and Tanzania (north). The east side comprises a coastline (Indian Ocean) [26]. The capital, which is located on the southern coast, is Maputo City, and the official language is Portuguese [27].

The country is divided in 11 provinces distributed through three regions: North (3 provinces), center (4 provinces) and south (4 provinces) [26]. Mozambique is endemic to tropical infectious diseases including malaria, yellow fever and waterborne maladies such as rotavirus and cholera [8]. As a low-income country, its population faces poverty and food insecurity, and the government faces financial constraints severely compromising the healthcare system [29]. Furthermore, areas surrounding major cities (e.g., Maputo, Matola, Beira, Nampula and Nacala) are densely populated but the sanitation system is deficient, contributing to cases of infectious maladies [30].

## 4. Overview of Cyclone Kenneth

According to the United Nations Office for the Coordination of Humanitarian Affairs (OCHA), Cyclone Kenneth originated in the Indian Ocean, and it was the strongest in the recorded history of the African continent [21]. It was also the first time Mozambique was hit by two major cyclones (Idai and Kenneth) in the same season [31]. Kenneth passed north Comoros Islands on 24 April and hit the island of Ngazidja (Grande Comore), resulting in 1000 displacements, 20 people injured and four deaths [21,31]. It entered northern Mozambique on the evening of 25 April 2019, destroying approximately 40,000 houses, 19 heath facilities, and leaving 374,000 people in need for assistance [21,32,33]. The date corresponded to the end of the rainy season, thus a cholera outbreak was regarded as highly probable [6].

The initial displacement due to Kenneth was 18,029 people but it increased substantially as the cyclone moved through the area, and at least 45 people died [21]. For instance, in Matemo Island (Ibo district), over 85% or the 3000 residents lost their houses [32]. The amount needed to assist the victims of Kenneth in total was US $104 million, and it is known that the United Nation’s Central Emergency Response Fund (CERF) offered at least US $13 million to support both Comoros and Mozambique [21,31]. Up to 6 May 2019, 32.6% of the total amount required had been funded [34].

## 5. Epidemiological Accounts

**Figure 2:**
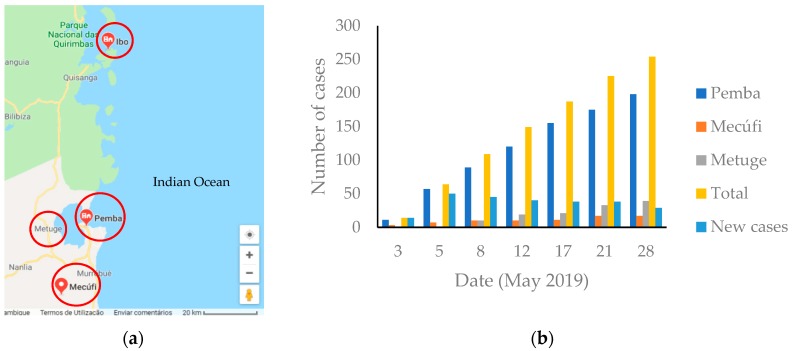
Summary of epidemiological information just after the declaration of the outbreak up to 28 May 2019: (**a**) focal areas of cholera control interventions and (**b**) number of cases of cholera. Sources: Both the map and chart were based on OCHA flash updates on Cyclone Kenneth [2,5,22], Agence France-Presse [6], and the National Health Institute and World Health Organization [17,33,35,36]. The map was adapted from Google Maps^TM^ (search term: Cabo Delgado Province).

Figure 2 shows priority areas for intervention and how the number of confirmed cases of cholera increased over time. Attention was focused to four areas, based on the population density, proximity to Pemba (Mecúfi and Metuge) and previous history of outbreaks. As the capital of Cabo Delgado, Pemba is the area where people live in closer contact with each other, and this can facilitate transmission of *Vibrio cholerae*, the bacterium responsible for cholera. Indeed, Pemba consistently presented the highest number of cases, and notably the highest transmission rate and also cumulative attack rate by 28 May 2019 (98.7 per 100,000 inhabitants) [35]. Metuge took longer to register the first cases but within a week surpassed Mecúfi. Ibo did not register any case, and it might have been due to its distance from Pemba, and its isolation as an island, besides the outbreak response.

Although the number of cases increased substantially, the number of new cases seemed to start declining after 5 May. It is perhaps related to an efficient response, following guidelines of the 2018–2019 Mozambique Humanitarian Response Plan [37], already prepared and revised after cyclone Idai. Yet, it is important to bear in mind that cyclone Kenneth added to the challenges after the devastation of Idai, though the experience from the first cyclone was certainly an asset for the management of the second.

In the 4th National Situation Report, INS stated that no fatality due to cholera had been recorded up to 31 May 2019 [35]. This fact is not surprising, considering that the 2015 series of outbreaks in the center-north of Mozambique registered a fatality rate of 0.7% [13]. This fatality rate is low considering the observed fatality rate of 0–15.8% from 22 countries with the highest incidence of cholera [38]. Cholera is highly virulent but also easy to treat [39]. A vaccination campaign started on 16 May and it might have further contributed to reducing the incidence.

There is little information currently available about the effectiveness of the response. Thus, further investigation is required. Cholera is just part of the calamity, but there were other concerns such as malaria, food insecurity, people who lost their houses, relatives, and much more [4,5].

## 6. Outbreak Response

The response was led by the Ministry of Health (MINED), represented by Cabo Delgado’s Provincial Directorate. The target areas for response were Pemba city, Macomia Town, and the Quissanga and Ibo islands. In these areas, the government (MINED) and partners such as OCHA and Médecins Sans Frontières (MSF) adapted the Cholera Response Plan for Beira after cyclone Idai [5,37]. The plan has four major health-related response procedures: (1) social mobilization campaigns for prevention, (2) establishment of treatment centers and units, (3) coordination to ensure improvement of water, sanitation and hygiene (WASH) and (4) surveillance.

The government and partners were already taking action to prevent cholera cases prior to the outbreak announcement. For instance, a team of epidemiologists, public health specialists and logisticians from the WHO, working in Beira city, was requested to assess the impact of cyclone Kenneth on public health in Cabo Delgado [5,32]. As of 1 May 2019, they were planning social mobilization campaigns [4]. Two days later, in Macomia Town, the teams were in the field to interact with the community, and the following day they started instructing in schools and hospitals how to disinfect wells [5]. Then, the training was scaled-up to all affected districts, and messages were aired through community radios to victims of the cyclone [22]. In parallel, the National Health Institute (INS), together with the WHO and Centers for Disease Control and Prevention (CDC) organized a short course on outbreak investigation, on 9 and 10 May 2019 [17]. The following step was to map the neighborhoods with community mobilizers to support hygiene promotion activities [22]. There was a short course on cholera case management for selected nurses from prioritized districts, from 27 to 29 May 2019 in Pemba, organized by COSACA (a consortium of Save the Children, Oxfam and Care) [35].

A Cholera Treatment Center had been established in Pemba city by 2 May 2019, with an initial capacity of 50 beds [2,4,17]. Later, another center was opened in Mecúfi (16 beds) [17], and minor units started operating throughout both areas [2,5]. On 5 May 2019, OCHA reported the distribution of cholera kits to Mecúfi treatment units [22]. Then, one more center was open in Pemba and Metuge (20 beds), and all treatment centers started receiving health education messages through radio [17,22]. On 12 May, the Ministry of Health delivered over 516,000 doses of Oral Cholera Vaccine (OCV) to Cabo Delgado’s local government and partners. The quantity was enough to deliver two doses (rounds) per person. The first round of immunization campaign would be between 16 and 20 May 2019 in Pemba city and between 17 and 21 May in Mecufi and Metuge [1,2,17,33]. By 26 May 2019, 252,448 people were successfully vaccinated [40].

According to Anjichi-Kodumbe et al. [4,5], as the outbreak was declared, initial WASH interventions consisted of distributing 120 bottles of Certeza^TM^ (chlorine water sanitizer [41]) for 600 people in Ibo, 30,000 to Pemba, and 5000 bars of soap to Pemba. Just after that, the intervention team engaged in supporting the treatment of wells to improve their safety in Ibo and preparing food to provide to the victims of the cyclone in two accommodation centers in Pemba (Desportivo and Centro do Congresso), in collaboration with the Swiss Agency for Development and Cooperation (SDC) [5,22]. Such centers ended up overcrowded and with suboptimal sanitary conditions, but effort was done to improve the situation [34]. Such effort included the distribution of plastic sheeting, water buckets, tents [42] and hygiene kits from the United Nations Population Fund (UNFPA) [22], with underwear, bath soap, toothbrush and toothpaste, sanitary napkins, washing powder, comb, flashlight and a reusable menstrual pad set [43]. In Macomia, schools and hospitals received water trucking, while in Ibo and Pemba received more bottles of Certeza^TM^ (4000 and 7000, respectively) [22]. Mapping the neighborhoods made it easier to systematically distribute the bottles [22]. In total, the WASH team distributed 48,000 bottles of water sanitizer [40].

INS, WHO and CDC combined their efforts to support the implementation of the Early Warning, Alert and Response System (EWARS), preparing local surveillance technicians for outbreak investigation in the districts [4,17]. The reports do not explain the extent at which each organization participated in the implementation of EWARS but, since they were all supporting the local government, perhaps they prepared modules for an integrated course, and then the government managed EWARS with their assistance. It is also possible that INS directly managed EWARS as the government’s representative.

## 7. Remarks

The outbreak of cholera due to cyclone Kenneth was well-managed, considering the magnitude of the calamity. For instance, the rainy season in 2015 did not result in major infrastructure damage but the resulting series of outbreaks seemed more widespread and severe, causing 53 deaths [13]. The outbreak due to Kenneth was not as severe likely due to experience acquired from the previous outbreaks, and the efficient synergy between the government and other organizations that helped mobilize the response, particularly in healthcare assistance and services of sanitation and hygiene [40]. As the time goes by, there have been efforts to better understand the epidemiology of cholera, and it is also expected to improve the way Mozambique responds to outbreaks. For instance, research organizations such as the National Health Institute (INS) or the Manhiça Health Research Center (CISM) have been highly engaged in studying and improving the strategies to control cholera in the country, fully assessing its epidemiology and risk factors and proposing solutions.

Not less important was the collaboration of the citizens, certainly motivated by the traumatic experience during the landing of cyclone Kenneth. This fact cannot be underestimated because previous interventions to control cholera throughout the country faced people’s unwillingness to collaborate, especially in northern Mozambique, because of conspiracy theories stating that the government maliciously introduced cholera to harm the population [44,45,46]. In 2016, Victorino et al. [46] interviewed 30 adults in three neighborhoods of Nampula city and they unanimously blamed the government for the cholera outbreak. The authors attributed such misunderstanding to limited knowledge on what really causes the disease and how it is transmitted, and rain is a common phenomenon that they might not associate with increased risk of exposure to *Vibrio cholerae* O1, and most might not even know this pathogen exists. In Gurúè city, central Mozambique, some people believe witch doctors cast cholera as a spell onto others. However, after a tragedy like cyclone Kenneth, people accepted more naturally the aftermath and the losses certainly left few options to the victims other than collaborating with the authorities.

If future calamites like cyclones Idai or Kenneth occur, there shall be at least the same level of commitment between the government, NGOs and the population to control cholera and other diarrheal diseases as it happened this year. Yet, it is important to be realistic and prepare strategies to accommodate scenarios in which there is insufficient financial assistance, as it initially occurred during cyclone Idai [19]. It would be a good idea for the government to maintain a contingency fund enough for such kind of emergencies.

As final comments, it is worth discussing why there were two cyclones within the same year in Mozambique after several decades without such kind of phenomena, despite some episodes of flood during the rainy season. It would be a good idea to explore the possibility of some connection between these cyclones and climate change, as some simulation-based studies have been presenting evidence [47,48,49]. According to Walsh et al. [49], climate change is resulting in stronger storms and increasing rainfall rates, although the number of cyclones is actually decreasing. If climate change is causing the cyclones, or at least affecting their strength and impact, it is certainly one more way in which it increases dissemination of waterborne diseases, including cholera. Some publications present a significant association between climate change and waterborne maladies [50,51,52,53], and it makes sense, as diseases such as cholera and malaria are highly affected by heavy rain, especially in suburban areas where there is improper sanitation. Studies associating the recent cyclones with global warming can be useful to predict the risk of the next cyclones and better address the issues resulting from the calamities, including the management of potential outbreaks of infectious diseases such as malaria and cholera. In the case of Mozambique, as storms are becoming potentially more frequent due to climate change, the government needs to prepare effective, proven response plans to combat outbreaks of waterborne diseases resulting from cyclones.
